# Placebo-controlled study with OHIO chamber of prophylactic pranlukast for children with Japanese cedar pollinosis: TOPIC-J III study

**DOI:** 10.3109/21556660.2014.960969

**Published:** 2014-09-03

**Authors:** Kei Hosoya, Satoru Masuno, Kazuhiro Hashiguchi, Kimihiro Okubo

**Affiliations:** 1 Department of Otorhinolaryngology, Nippon Medical School, TokyoJapan; 2 Otorhinolaryngology, Futaba Clinic, TokyoJapan

**Keywords:** Cedar pollinosis, Pranlukast, Nasal symptoms, Pediatric patients, Prophylactic therapy

## Abstract

**Objective:**

This double-blind, placebo-controlled comparative study was designed to investigate whether pranlukast dry syrup, a leukotriene receptor antagonist, has a protective effect against priming, controlled pollen exposure, and natural pollen exposure in children with Japanese cedar pollinosis.

**Research design and methods:**

Thirty children aged 12–15 years with Japanese cedar pollinosis (positive skin test for Japanese cedar pollen), who had suffered from pollinosis for at least 2 years and developed severe nasal obstruction when exposed to Japanese cedar pollen, were enrolled in this study. They were randomly allocated to treatment with pranlukast or placebo orally after breakfast and dinner for 8 weeks during the Japanese cedar pollen season. Soon after the start of the pollen season, all subjects underwent a challenge by exposure for 3 h to Japanese cedar pollen (8000 grains/m^3^) in an artificial exposure chamber (OHIO chamber).

**Clinical trial registration:**

The University Hospital Medical Information Network in Japan (UMIN000009840).

**Main outcome measures:**

The effect of pranlukast was evaluated using self-rating of nasal symptoms by the subjects and measurement of eosinophil cationic protein in nasal discharge specimens.

**Results:**

Scores for the symptoms of pollinosis were lower in the pranlukast group than in the placebo group during treatment in the priming state, as well as after controlled pollen exposure and natural pollen exposure. Pranlukast significantly improved the score for nasal obstruction, compared with placebo. A correlation was found between changes of the scores for symptoms of pollinosis and changes of the eosinophil cationic protein level.

**Conclusions:**

These results confirm a protective effect of pranlukast against both priming and challenge (controlled and natural) with Japanese cedar pollen. The present findings suggested that pranlukast dry syrup may be useful for prophylaxis against pollinosis in children.

## Introduction

Japanese cedar (JC) pollinosis is a form of seasonal allergic rhinitis peculiar to Japan that was first reported in 1964^1^. It is caused by pollen dispersal in the early spring from Japanese cedar and cypress trees throughout Japan, which release a massive amount of pollen. In Japan, the prevalence of JC pollinosis increased by ∼10% over 10 years from 16.2% in 1998 to 26.5% in 2008^2,3^. In 2008, the prevalence of JC pollinosis was 33.1–39.1% among adults in each decade from the 20s to the 50s, 31.4% in teenagers and 13.7% in children aged 5–10 years^4^. The prevalence of JC pollinosis in children appears to be much higher than that of allergic rhinitis, which was reported as 10% or less in children aged 6–7 and ∼15% in those aged 13–14 years^5,6^.

In Japan, the pollen season usually starts in early February and ends between mid-March and early April. JC pollinosis causes nasal and ocular symptoms that can significantly influence the quality-of-life (QoL)^7^, and it is important to initiate treatment before or at the start of the pollen season to prevent the development of symptoms. Accordingly, the Japanese guideline for nasal allergy recommends initiation of prophylactic therapy before or early in the pollen season^4^.

Leukotrienes dilate vessels in the nasal mucosa^8^, increase vascular permeability^9^, and promote the migration of inflammatory cells such as eosinophils and macrophages^10–12^. Thus, leukotrienes are important chemical mediators of allergic rhinitis, suggesting that usefulness of leukotriene receptor antagonists (LTRAs)^13–15^. Clinical studies have shown that LTRAs are as effective as antihistamines for pollinosis^16–18^. It has also been reported that initiating prophylactic LTRA therapy before or early in the pollen season can prevent symptoms of pollinosis^19–22^.

The LTRA pranlukast is effective for perennial allergic rhinitis in adults^23^. It is also superior to placebo as prophylactic therapy for pollinosis^21,22^. Furthermore, pranlukast has an immediate effect against exposure to pollen in an artificial exposure chamber (OHIO chamber), improving the early and late phases of nasal obstruction^24^, and inhibits priming for pollinosis^25^. Moreover, pranlukast dry syrup (PLK-DS) is more effective than placebo for preventing nasal obstruction in children with pollinosis tested in an OHIO chamber^26,27^.

Accordingly, this study was performed to determine the efficacy of prophylactic therapy with PLK-DS vs placebo for JC pollinosis in children by investigating the inhibitory effect on priming for pollinosis and on the response to pollen exposure (both controlled exposure in an OHIO chamber and natural exposure during the pollen season).

## Materials and methods

### Subjects

This study enrolled 30 children with JC pollinosis who met the following inclusion criteria: aged from 10 years to younger than 15 years, class 2–6 when multiple antigen testing was performed to detect specific IgE antibodies for JC pollen in serum, a history of nasal symptoms with nasal obstruction following exposure to JC pollen in an OHIO chamber, development and exacerbation of nasal symptoms with nasal obstruction during the annual JC pollen season, and those determined to be eligible by an investigator or sub-investigator when clinical examination was done before the start of the study. The exclusion criteria were as follows: any lesion of the nasal or ocular mucosa, use of parenteral corticosteroids within 6 months before obtaining informed consent, nasal disease (deviation of the nasal septum, nasal polyps, etc.), systemic disease (asthma, tuberculosis, etc.), a history of anaphylaxis, a history of hypersensitivity to any ingredient of the test drug, current immunotherapy (hyposensitization therapy or allassotherapy), and any other reason that made patients ineligible for this study according to the investigator. We specified two persons who were responsible for patient enrollment (K.H. and S.M). It was also pre-determined that if they could not reach a consensus as to whether to include/exclude a child, an independent researcher (Dr Minoru Gotoh, Nippon Medical School Tama Nagayama Hospital, Tokyo, Japan) would be consulted to make the final decision.

The subjects received a detailed explanation of this study and possible adverse reactions to the test drug, and their parents or guardians provided informed consent. The study was performed at Medical Corporation Shinanokai Samoncho Clinic according to the ethical guidelines in the protocol and the Declaration of Helsinki (2000) after receiving approval from the ethics committee of the clinic. This study was registered with the University Hospital Medical Information Network in Japan (registration No. UMIN000009840).

### Study procedures

**Figure 1.**
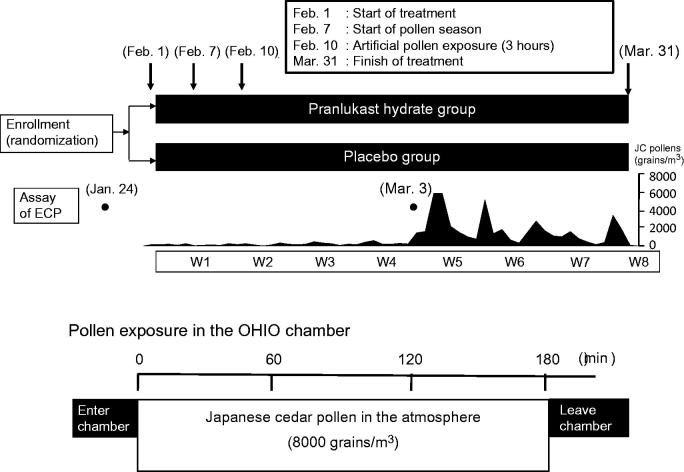
Study schedule. ECP, eosinophil cationic protein; JC, Japanese cedar.

This was a randomized, double-blind, placebo-controlled clinical trial. The investigational drugs included PLK-DS (10% pranlukast) and placebo, which were confirmed to be indistinguishable from each other. In late January 2013, 30 participants were selected based on the results of skin tests with JC pollen. For the skin test, a skin prick test was performed. When ‘the test site showed a wheal of at least 5 mm surrounded by at least 10 mm of erythema’, the judgment was positive. According to the schedule shown in Figure 1, the participants received PLK-DS or placebo twice a day (after breakfast and after dinner) for 2 months from February 1, which was expected to be before the start of the JC pollen season, through the peak pollen season until March 31.

‘The starting date of the pollen season’ is defined as the day when at least 1 pollen grain/cm^2^ has been detected for at least 2 days by an automatic pollen measuring device (Durham pollen gathering device) at the Ministry of the Environment. In fact, a very low level of pollen dispersion occurs before the official start of the pollen season, and some patients develop symptoms before the starting date.

Early in the JC pollen season (February 10), subjects were exposed to JC pollen for 3 h in an OHIO chamber (JC pollen count: 8000 grains/m^3^)^28–30^. Before the JC pollen season (January 24) and during the pollen season (March 3), eosinophil cationic protein (ECP) was measured in nasal discharge samples as an index of inflammation. Extraction of ECP from nasal discharge samples and measurement were done according to the manufacturer’s recommendations (MESACUP ECP Test: Medical & Biological Laboratories Co., Ltd., Aichi, Japan)^27^. Concomitant use of the following drugs was not permitted during treatment with the investigational drug: corticosteroids (oral, inhaled, and topical), antihistamines (oral, inhaled, and topical), anti-allergic drugs, leukotriene antagonists, anti-prostaglandin D_2_/thromboxane A_2_ drugs, vasoconstrictors, anticholinergics, and other drugs that could affect evaluation of efficacy. However, if a patient developed severe symptoms following the start of the pollen season, rescue medications could be used to treat the symptoms (including Intal Nasal Spray 2%, Intal Ophthalmic Solution 2%, vasoconstrictors, and anticholinergics), provided that the total nasal symptoms score (Table 1) was ≥4 points on the previous day. As rescue medication for severe symptoms while patients were in the OHIO chamber or after leaving the chamber, Intal Nasal Spray 2% and/or Intal Ophthalmic Solution 2% could be used if this was determined to be necessary by an investigator or sub-investigator. Atmospheric JC pollen levels were based on the data published by Nippon Medical School.

**Table 1.** Nasal symptoms questionnaire.

Sneezing – ‘How severe is sneezing?’
0: None/1: Mild/2: Moderate/3: Severe/4: Very severe
Nasal discharge – ‘How severe is running nose?’
0: None/1: Mild/2: Moderate/3: Severe/4: Very severe
Nasal obstruction – ‘How severe is nasal obstruction?”
0: None/1: Mild/2: Moderate/3: Severe/4: Very severe

### Evaluation

Nasal symptoms were recorded in the nasal allergy diary before sleeping every day from February 1 to March 31. On the day after controlled pollen exposure in the OHIO chamber, symptoms were also recorded in the morning and at noon. The most severe symptoms during each period were recorded in the diary.

In the nasal allergy diary, the severity of each symptom (sneezing, nasal discharge, and nasal obstruction) was assigned a score from 0–4^24^. The weekly mean scores were calculated from February 1 for each symptom and for all three symptoms combined (total score), and changes from the mean score of Week 1 (including the first day of the pollen season and before controlled exposure in the OHIO chamber) were determined for each of the subsequent weeks. ECP levels were compared at two times, which were before the start of the JC pollen season and during the pollen season.

### Statistical analysis

Data were analyzed with a two-sided U-test and the Wilcoxon test, and *p* ≤ 0.05 was accepted as the level of significance. Statistical analysis was performed using SAS version 8.02 (SAS Institute Inc., Cary, NC).

## Results

### JC pollen season

The JC pollen season started on February 7 (7 days after commencing treatment) (Figure 1). In February, the daily JC pollen count was relatively low. However, the count increased during Week 5 and was at least 2000 grains/m^3^ in each week up to Week 8 (end of the study).

### Patient demographic profile

Of the 30 patients enrolled, 25 completed this study and five were excluded due to insufficient data in the nasal allergy diary (Table 2). Among the 25 patients, 13 received PLK-DS (six boys and seven girls; mean age: 13.77 ± 1.17 years) and 12 received placebo dry syrup (four boys and eight girls; mean age: 13.67 ± 0.98 years). There was no significant difference of nasal symptom scores between the two groups in Week 1, and both groups had mild symptoms at that time (Table 3).

**Table 2.** Patient characteristics at baseline.

	Pranlukast hydrate group	Placebo group
No. of patients	13	12
Sex
Male	6 (46.2%)	4 (33.3%)
Female	7 (53.8%)	8 (66.7%)
Age, years
12	3 (23.1%)	1 (8.3%)
13	1 (7.7%)	5 (41.7%)
14	5 (38.5%)	3 (25%)
15	4 (30.8%)	3 (25%)
Mean ± SD	13.77 ± 1.17	13.67 ± 0.98
CAP-RAST
Class 3	0	1 (8.3%)
Class 4	0	1 (8.3%)
Class 5	1 (7.7%)	1 (8.3%)
Class 6	12 (92.3%)	9 (75%)

**Table 3.** Mean symptom scores in the first before pollen exposure in the OHIO chamber.

	Pranlukast hydrate group (mean ± standard error)	Placebo group (mean ± standard error)	U-test
Sneezing	0.582 ± 0.222	0.583 ± 0.205	*p* = 1.000
Rhinorrhea	1.110 ± 0.255	0.893 ± 0.248	*p* = 0.566
Nasal obstruction	1.011 ± 0.261	0.667 ± 0.239	*p* = 0.393
Total nasal symptoms	2.703 ± 0.603	2.143 ± 0.606	*p* = 0.369

### Nasal symptoms

#### Sneezing

**Figure 2.**
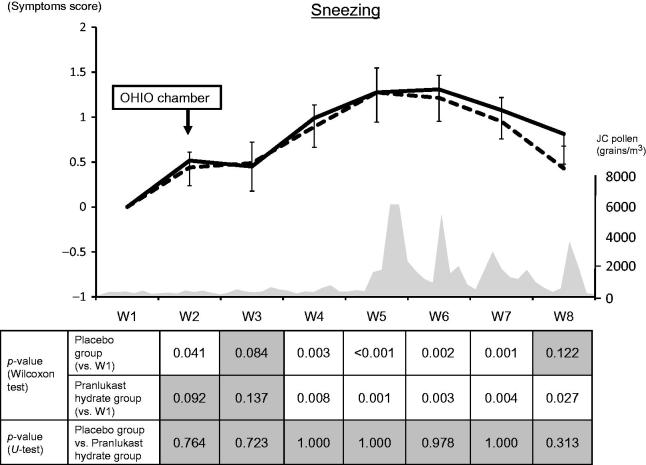
Weekly mean sneezing scores. The solid line and dashed line represent the pranlukast hydrate group and the placebo group, respectively. 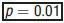 : significant, 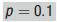 : not significant. JC, Japanese cedar.

In Week 2, subjects were exposed to JC pollen in the OHIO chamber. Subsequently, the sneezing score increased significantly in the placebo group, while there was no significant increase in the PLK-DS group. From Week 3 to Week 7, the sneezing score showed a significant increase in both groups (Figure 2).

#### Nasal discharge

**Figure 3.**
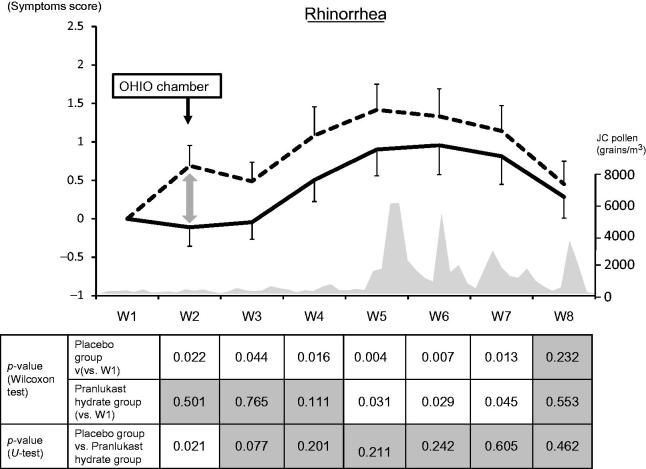
Weekly mean rhinorrhea scores. The solid line and dashed line represent the pranlukast hydrate group and the placebo group, respectively. 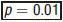 : significant, 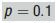 : not significant. JC, Japanese cedar.

In Week 2 (OHIO chamber), the nasal discharge score increased significantly in the placebo group, while there was no significant increase in the PLK-DS group. In addition, the placebo group had a significantly higher nasal discharge score compared with the PLK-DS group. In Weeks 3 and 4 (before the peak of the pollen season), there was a significant increase of the score in the placebo group, but not in the PLK-DS group. Between Weeks 5 and 7 (peak pollen season), a significant increase of the score was observed in both groups (Figure 3).

#### Nasal obstruction

**Figure 4.**
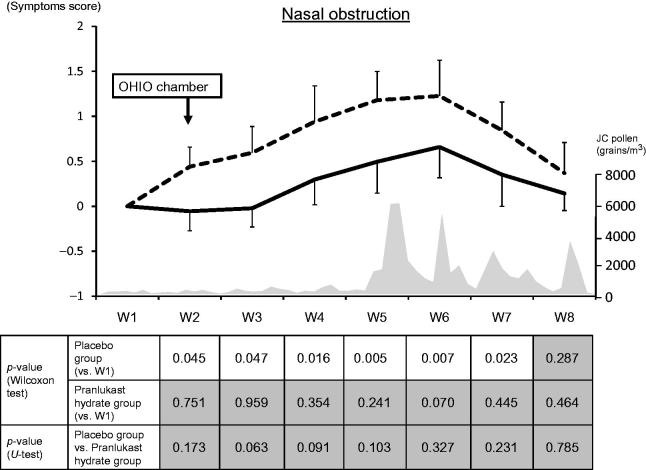
Weekly mean nasal obstruction scores. The solid line and dashed line represent the pranlukast hydrate group and the placebo group, respectively. 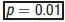 : significant, 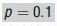 : not significant. JC, Japanese cedar.

In the placebo group, a significant increase of the nasal obstruction score continued from Week 2 (OHIO chamber) to Week 7 of the JC pollen season. On the other hand, there was no significant increase of the score in the PLK-DS group from Week 2 to Week 8 (end of the study) (Figure 4).

#### Total score

**Figure 5.**
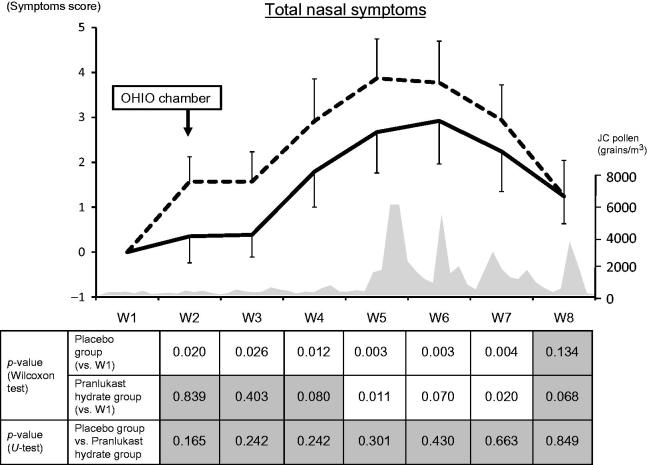
Weekly mean total nasal symptoms scores. The solid line and dashed line represent the pranlukast hydrate group and the placebo group, respectively. 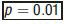 : significant, 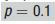 : not significant. JC, Japanese cedar.

In the placebo group, the total nasal symptoms score was significantly increased between Week 2 (OHIO chamber) and Week 7 (peak pollen season). In the PLK-DS group, there was no significant increase between Week 2 and Week 4 (before the peak pollen season), although a significant increase of the score was observed during Weeks 5–7 (peak pollen season) (Figure 5).

### Changes of ECP

**Figure 6.**
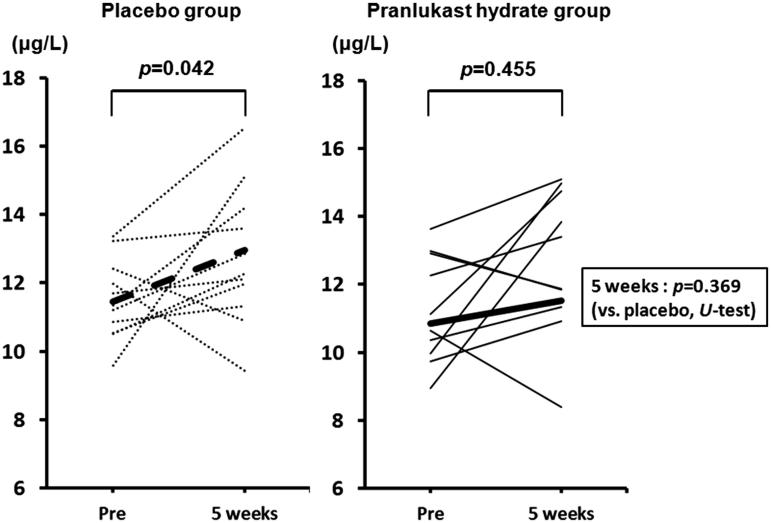
Changes of eosinophil cationic protein.

In the placebo group, the ECP level in nasal discharge increased significantly from 11.46 ± 0.33 μg/L before the start of the pollen season (January 24) to 12.97 ± 0.60 μg/L during the peak pollen season (March 3) (*p* = 0.042, Wilcoxon test). On the other hand, the PLK-DS group showed no significant increase of ECP in nasal discharge (from 10.86 ± 0.89 μg/L to 11.52 ± 1.08 μg/L; *p* = 0.455, Wilcoxon test) (Figure 6).

### Use of rescue drugs

Rescue drugs were hardly used during the early pollen season (W1–4). The number of days when a dosage of 0.5 drops/day was exceeded was 0 days for Intal Nasal Solution 2% in both the placebo group and the pranlukast dry syrup group. For Intal Ophthalmic Solution 2%, a dosage of 0.5 drops/day was exceeded on 0 days in the placebo group and 1 day in the pranlukast dry syrup group. During the late pollen season (W5–8), there was a slight increase in use, with a dosage of 0.5 drops/day, for Intal Nasal Solution 2% being exceeded on 0 days in the placebo group and 1 day in the pranlukast dry syrup group. For Intal Ophthalmic Solution 2%, the dosage of 0.5 drops/day was exceeded on 2 days in the placebo group and 3 days in the pranlukast dry syrup group.

## Discussion

The main findings of the present study were as follows: First, prophylactic administration of PLK-DS to children with JC pollinosis demonstrated an inhibitory effect on priming in the early part of the pollen season. Second, prophylaxis with PLK-DS reduced the symptoms caused by exposure to JC pollen in an OHIO chamber during the early part of the pollen season. In particular, PLK-DS prophylaxis reduced nasal discharge and nasal obstruction after controlled exposure to JC pollen in the OHIO chamber. Third, prophylaxis with PLK-DS inhibited an increase of ECP in nasal discharge specimens during the peak pollen season. These findings indicated that PLK-DS prophylaxis was effective for reducing symptoms of pollinosis and nasal inflammation in children with JC pollinosis.

We previously reported that PLK-DS was effective in children who were exposed to pollen using the OHIO chamber^26^. In that study, we measured ECP levels in nasal discharge specimens at the end of pollen exposure, and found that PLK-DS inhibited an increase of ECP^27^. Although we demonstrated that PLK-DS suppressed inflammation and pollinosis symptoms, that study was not performed during the pollen season and did not confirm the effect of the drug on natural exposure to pollen. Also, prophylactic therapy is important in children as well as in adults, hence we considered it important to determine the efficacy of PLK-DS prophylaxis in children with pollinosis.

The pathogenesis of allergic rhinitis is considered to involve minimal chronic inflammation^31,32^. It is thought that repeated exposure to low levels of antigens causing no symptoms leads to chronic inflammation of the nasal mucosa. In fact, repeated out-of-season exposure of patients with seasonal allergic rhinitis to 1/100 of the antigen dose causing symptoms has been shown to increase histamine and ECP levels in nasal discharge^33^. It was also reported that symptom returned to baseline by 6 weeks after the pollen season, but the levels of interleukin-1, leukotriene, and ECP in nasal discharge remained high^34^. Assuming that patients have minimal chronic inflammation, we consider that initiation of prophylactic therapy with anti-inflammatory agents either before or early in the pollen season is a logical method to reduce the deterioration of symptoms and QoL during the pollen season.

The present placebo-controlled study demonstrated that PLK-DS prophylaxis is useful for children with pollinosis. As has been reported in adults^20–22^, the nasal symptoms scores (especially for nasal obstruction) of the PLK-DS group were significantly lower than those of the placebo group. Since use of PLK-DS as prophylactic therapy in children with pollinosis delays the onset of nasal symptoms as it does in adults^22^ and, thus, delays the deterioration of QoL and the need for symptomatic medication, this drug appears to be appropriate for prophylaxis because of its anti-inflammatory effect.

A limitation of this study was the small sample size, which was probably the reason for the few significant differences between the PLK-DS and placebo groups. Because the maximum number of patients who could fit in the pollen exposure chamber used in this study was 15 and it was important to test all subjects under similar conditions during the pollen season, we limited the number of subjects for this study. In the future, a larger multi-center, placebo-controlled, double-blind study should be performed to confirm the usefulness of PLK-DS as prophylactic therapy for JC pollinosis in children. Another limitation was that the dropout rate was rather high. The five dropouts were due to insufficient entries in the nasal allergy diary. Patients were instructed to make daily entries in this diary before bedtime about the most serious symptoms of that day. The method of evaluation was to calculate the weekly mean score and determine the change from the mean score of the first week. If entries were missed, it could have been possible to calculate the mean value from the remaining data. However, the severity of pollinosis is highly dependent on the amount of pollen dispersed in the atmosphere, and this varies from day to day. We, therefore, considered it inappropriate to calculate the mean score when data were missing.

## Conclusion

In children with JC pollinosis, we demonstrated an inhibitory effect of PLK-DS on priming in the early pollen season, as well as inhibition of symptoms caused by controlled exposure to pollen and symptoms during the pollen season. PLK-DS was also shown to suppress an increase of ECP in nasal discharge, indicating an anti-inflammatory effect. The present findings suggested that pranlukast dry syrup may be useful for prophylaxis against pollinosis in children.

## References

[1] Horiguchi S, Saito Y Japanese cedar pollinosis in Nikko, Japan. Jpn J Allergol 1964;13:16-18 (in Japanese with English abstract) 14112320

[2] Okuda M Epidemiology of Japanese cedar pollinosis throughout Japan. Ann Allergy Asthma Immunol 2003;91:288-96 14533662 10.1016/S1081-1206(10)63532-6

[3] Sakashita M, Hirota T, Harada M, et al Prevalence of allergic rhinitis and sensitization to common aeroallergens in a Japanese population. Int Arch Allergy Immunol 2010;151:255-61 19786806 10.1159/000242363

[4] Okubo K, Kurono Y, Fujieda S, et al Japanese guideline for allergic rhinitis. Allergol Int 2011;60:171-89 21636965 10.2332/allergolint.11-rai-0334

[5] Asher MI, Keil U, Anderson HR, et al International study of asthma and allergies in childhood (ISAAC): rationale and methods. Eur Respir J 1995;8:483-91 7789502 10.1183/09031936.95.08030483

[6] The International Study of Asthma and Allergies in Childhood (ISAAC) Steering Committee. Worldwide variation in the prevalence of symptoms of asthma, allergic rhinoconjunctivitis and atopic eczema: ISAAC. Lancet 1998;351:1225–32 9643741

[7] Okubo K, Gotoh M, Shimada K, et al Fexofenadine improves the quality of life and work productivity in Japanese patients with seasonal allergic rhinitis during the peak cedar pollinosis season. Int Arch Allergy Immunol 2005;136:148-54 15650312 10.1159/000083322

[8] Mizutani N, Nabe T, Imai A, et al Markedly increased nasal blockage by intranasal leukotriene D4 in an experimental allergic rhinitis model: contribution of dilated mucosal blood vessels. Jpn J Pharmacol 2001;86:170-82 11459119 10.1254/jjp.86.170

[9] Shirasaki H, Kojima T, Asakura K, et al The pathophysiological role of kinin and chemical mediators on experimental allergic rhinitis. Adv Exp Med Biol 1989;247A:375-8 2603805 10.1007/978-1-4615-9543-4_56

[10] Oshima N, Nagase H, Koshino T, et al A functional study on CysLT(1) receptors in human eosinophils. Int Arch Allergy Immunol 2002;129:67-75 12373000 10.1159/000065175

[11] Thivierge M, Stankova J, Rola-Plerszczynski M IL-13 and IL-4 up-regulate cysteinyl leukotriene 1 receptor expression in human monocytes and macrophages. J Immunol 2001;167:2855-60 11509632 10.4049/jimmunol.167.5.2855

[12] Peters-Golden M, Gleason MM, Togias A Cysteinyl leukotriene: multi-functional mediators in allergic rhinitis. Clin Exp Allergy 2006;36:689-703 16776669 10.1111/j.1365-2222.2006.02498.x PMC1569601

[13] Fujita M, Yonetomi Y, Takeda H, et al Effects of specific cysteinyl leukotriene antagonist, pranlukast, on antigen-induced cysteinyl leukotriene-mediated rhinitis in guinea pigs. Jpn J Pharmacol 1997;75:347-53 9469640 10.1254/jjp.75.347

[14] Lipworth BJ Emerging role of antileukotriene therapy in allergic rhinitis. Clin Exp allergy 2001;31:1813-21 11737031 10.1046/j.1365-2222.2001.01202.x

[15] Meltzer EO Clinical evidence for antileukotriene therapy in the management of allergic rhinitis. Ann Allergy Asthma Immunol 2002;88(4 Suppl 1):23-9 11991547 10.1016/s1081-1206(10)62025-x

[16] Philip G, Malmstrom K, Hampel FC, et al Montelukast for treating seasonal allergic rhinitis: a randomized, double-blind, placebo-controlled trial performed in the spring. Clin Exp Allergy 2002;32:1020-8 12100048 10.1046/j.1365-2222.2002.01422.x

[17] Van Adelsberg J, Philip G, Pedinoff AJ, et al Montelukast improves symptoms of seasonal allergic rhinitis over a 4-week treatment period. Allergy 2003;58:1268-76 14616102 10.1046/j.1398-9995.2003.00261.x

[18] Meltzer EO, Philip G, Weinstein SF, et al Montelukast effectively treats the nighttime impact of seasonal allergic rhinitis. Am J Rhinol 2005;19:591-8 16402647

[19] Kurowski M, Kuna P, Gorski P Montelukast plus cetirizine in the prophylactic treatment of seasonal allergic rhinitis: influence on clinical symptoms and nasal allergic inflammation. Allergy 2004;59:280-8 14982509 10.1046/j.1398-9995.2003.00416.x

[20] Sasaki K, Okamoto Y, Yonekura S, et al Cedar and cypress pollinosis and allergic rhinitis: quality of life effects of early intervention with leukotriene receptor antagonists. Int Arch Allergy Immunol 2009;149:350-8 19295239 10.1159/000205581

[21] Yonekura S, Okamoto Y, Okubo K, et al Beneficial effects of leukotriene receptor antagonists in the prevention of cedar pollinosis in a community setting. J Investig Allergol Clin Immunol 2009;19:195-203 19610262

[22] Gotoh M, Suzuki H, Okubo K Delay of onset of symptoms of Japanese cedar pollinosis by treatment with a leukotriene receptor antagonist. Allergol Int 2011;60:483-9 21778814 10.2332/allergolint.10-OA-0285

[23] Ueda T, Takeno S, Furukido K, et al Leukotriene receptor antagonist pranlukast suppresses eosinophil infiltration and cytokine production in human nasal mucosa of perennial allergic rhinitis. Ann Otol Rhinol Laryngol 2003;112:955-61 14653364 10.1177/000348940311201107

[24] Endo S, Gotoh M, Okubo K, et al Trial of pranlukast inhibitory effect for cedar exposure using an OHIO chamber. J Drug Assess 2012;1:48-54 10.3109/21556660.2012.703630 PMC4980729 27536428

[25] Okubo K, Hashiguchi K, Masuno S. Protective effect of pranlukast hydrate against the priming states of Japanese cedar pollen and the large of pollen-exposure. 17th Congress of the APSR (Asian Pacific Society of Respirology), Hong Kong, 2012

[26] Wakabayashi K, Hashiguchi K, Kanzaki S, et al Pranlukast dry syrup inhibits symptoms of Japanese cedar pollinosis in children using OHIO Chamber. Allergy Asthma Proc 2012;33:102-9 22370535 10.2500/aap.2012.33.3517

[27] Gotoh M, Okubo K, Hashiguchi K, et al Noninvasive biological evaluation of response to pranlukast treatment in pediatric patients with Japanese cedar pollinosis. Allergy Asthma Proc 2012;33:459-66 23394502 10.2500/aap.2012.33.3615

[28] Hashiguchi K, Tang H, Fujita T, et al Preliminary study on Japanese cedar pollinosis in an artificial exposure chamber (OHIO Chamber). Allergol Int 2007;56:125-30 17384532 10.2332/allergolint.O-06-453

[29] Hashiguchi K, Tang H, Fujita T, et al Pilot study of Japanese cedar pollen exposure using a novel artificial exposure chamber (OHIO Chamber). Clin Exp Allergy Rev 2008;8:30-6

[30] Hashiguchi K, Tang H, Fujita T, et al Validation study of the OHIO Chamber in patients with Japanese cedar pollinosis. Int Arch Allergy Immunol 2009;149:141-9 19127071 10.1159/000189197

[31] Storms WW Minimal persistent inflammation, an emerging concept in the nature and treatment of allergic rhinitis: the possible role of leukotrienes. Ann Allergy Asthma Immunol 2003;91:131-40 12952106 10.1016/S1081-1206(10)62167-9

[32] Canonica GW, Compalati E Minimal persistent inflammation in allergic rhinitis: implications for current treatment strategies. Clin Exp Immunol 2009;158:260-71 19765020 10.1111/j.1365-2249.2009.04017.x PMC2792821

[33] Roquat A, Ihre E, van Hage-Hamsten M, et al Allergen-induced inflammation in the nose: a comparison of acute and repeated low-dose allergen exposure. Allergy 1996;51:42-8 8721527 10.1111/j.1398-9995.1996.tb04548.x

[34] Bachert C, van Kempen M, van Cauwenberge P Regulation of proinflammatory cytokines in seasonal allergic rhinitis. Int Arch Allergy Immunol 1999;118:375-9 10224452 10.1159/000024141

